# Linking genes to diseases with a SNPedia-Gene Wiki mashup

**DOI:** 10.1186/2041-1480-3-S1-S6

**Published:** 2012-04-24

**Authors:** Benjamin M Good, Erik L Clarke, Salvatore Loguercio, Andrew I Su

**Affiliations:** 1 The Scripps Research Institute, 10550 North Torrey Pines Road, La Jolla, CA, 92037, USA; 2 Technische Universität Dresden, Biotechnology Center, Tatzberg 47/49, 01307 Dresden, Germany

## Abstract

**Background:**

A variety of topic-focused wikis are used in the biomedical sciences to enable the mass-collaborative synthesis and distribution of diverse bodies of knowledge. To address complex problems such as defining the relationships between genes and disease, it is important to bring the knowledge from many different domains together. Here we show how advances in wiki technology and natural language processing can be used to automatically assemble ‘meta-wikis’ that present integrated views over the data collaboratively created in multiple source wikis.

**Results:**

We produced a semantic meta-wiki called the Gene Wiki+ that automatically mirrors and integrates data from the Gene Wiki and SNPedia. The Gene Wiki+, available at (http://genewikiplus.org/), captures 8,047 distinct gene-disease relationships. SNPedia accounts for 4,149 of the gene-disease pairs, the Gene Wiki provides 4,377 and only 479 appear independently in both sources. All of this content is available to query and browse and is provided as linked open data.

**Conclusions:**

Wikis contain increasing amounts of diverse, biological information useful for elucidating the connections between genes and disease. The Gene Wiki+ shows how wiki technology can be used in concert with natural language processing to provide integrated views over diverse underlying data sources.

## Background

One of the key goals of current biomedical research is the elucidation of the relationships that hold between genes, environment and disease. Tackling this complex challenge requires the coordination of information emerging from a variety of scientific and medical communities. Increasingly, wiki technology is being used to enable such communities to collaboratively synthesize and distribute their knowledge. These ‘bio-wikis’ are emerging in many different areas [1,2]. We have wikis about genes [3-5], proteins [6], protein structures [7,8], SNPs [9], pathways [10], specific organisms [11] and many other biological entities. Bio-wikis have become important concept-centric knowledge resources but no single wiki contains all of the knowledge needed to answer most biological questions. The task of integrating the knowledge across different wikis remains the job of the end user. Recently, three key factors have emerged that make it possible to dynamically produce ‘meta-wikis’ that provide end users with consolidated views of information spanning multiple underlying wikis.

The first factor is the widespread adoption of the MediaWiki software for implementations within the bio-wiki community. MediaWiki installations now provide a powerful web API (Application Programming Interface) for direct, high-level access to the data contained in their databases. The API uses RESTful calls [12] to permit automated processes to make queries and post changes. Since many of the bio-wikis now have, by default, the same API, this implies that the same software can be used to query and edit the content of many different wikis without alteration.

The second factor is the increasing adoption of standardized systems for describing and recognizing biological concepts across multiple sites. Such systems provide identifiers for genes (e.g. NCBI Gene ids) and other biological concepts (e.g. Gene Ontology terms). These shared names can be used to identify when two different wikis contain knowledge pertinent to the same things and hence provide the key starting point for integration.

The third factor is the Semantic extension to the Media Wiki system [13]. By installing this extension, wiki administrators make it possible to add semantic links between articles in the wiki - for example, GeneX hasSNP snpY. These semantic links can be used in queries to the system that are much like queries to a database.

The combination of a consistent API across many different wikis, a growing collection of unifying ontologies and the Semantic extension enables the rapid formation of wiki-mashups or ‘meta-wikis’. Such meta-wikis offer the potential to produce integrated views of the knowledge dispersed across many different sources. Here we show how an automatically generated meta-wiki, called the Gene Wiki+, composed of elements drawn from SNPedia and the Gene Wiki exposes substantially more evidence of links between genes and diseases than either resource contains independently.

## Methods

### SNPedia

SNPedia provides textual information about links between variations in human genes and human phenotypes [9]. It uses standard identifiers from trusted authorities - primarily dbSNP [14] - to enable extensive linking to other public bioinfomatics databases and to personal genomics companies like 23andMe [15]. It is not a comprehensive listing of SNPs, rather it focuses on SNPs that have some evidence of association with a human phenotype.

### Gene Wiki

The Gene Wiki is an attempt to generate a collaboratively written, continuously updated review article for every human gene [4]. It provides textual descriptions of gene function in normal conditions as well as descriptions of the role the gene may play in disease. Currently, it includes more than 10,450 Wikipedia articles about human genes [16].

### Disease Ontology

The Human Disease Ontology was initiated in 2003 for the purpose of integrating biomedical data [17]. It is an open source, hierarchical controlled vocabulary of human diseases, now included as one of the OBO Foundry ontologies [18]. It is currently organized under 8 top-level categories: ‘disease by infectious agent’, ‘disease of anatomical entity’, ‘disease of cellular proliferation’, ‘disease of mental health’, ‘disease of metabolism’, ‘genetic disease’, ‘medical disorder’ and ‘syndrome’.

### SNPedia + Gene Wiki + Disease Ontology = Gene Wiki+

The Gene Wiki+ is a meta-wiki composed of information about genes drawn from the Gene Wiki, information about SNPs from SNPedia and Disease Ontology terms identified in both resources using the NCBO Annotator [19]. Bringing information about genes and diseases from both the Gene Wiki and SNPedia together into the Gene Wiki+ allows us to better address the following important question.

*“Based on what we know now*, *what genes are linked to which diseases?”*

**Figure 1:**
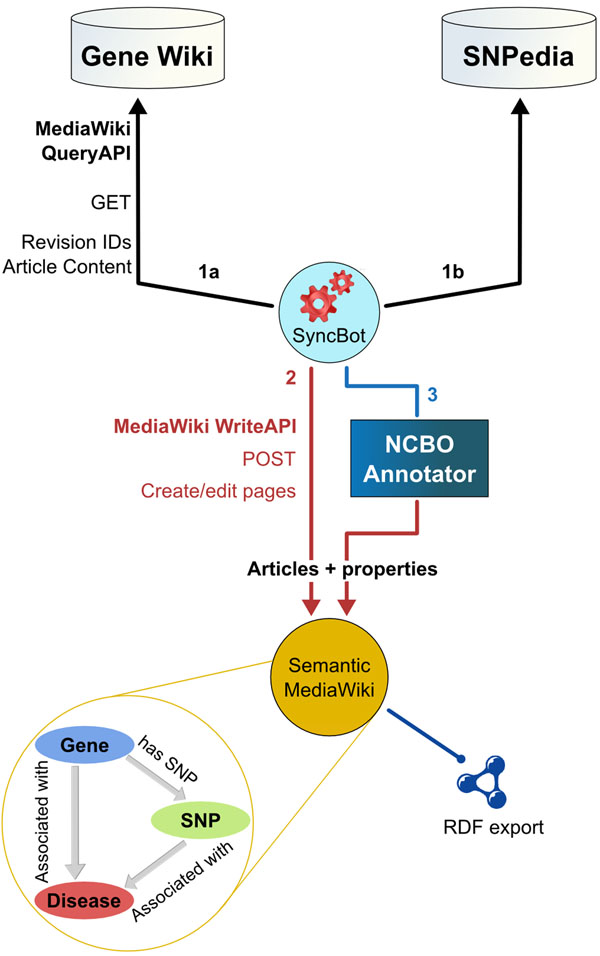
**The process of building the Gene Wiki+ meta-wiki**. The SyncBot mirrors articles from the Gene Wiki and SNPedia on a Semantic MediaWiki installation referred to as the Gene Wiki+. As part of the transfer process, terms from the Human Disease Ontology are identified in article text using the NCBO Annotator and used to form queriable semantic links.

We now describe how to automatically construct a semantic wiki instance suitable for exploring the relationship between genes and disease both by browsing and through structured queries. The resultant meta-wiki contains semantic relations linking genes to diseases, genes to SNPs, and SNPs to diseases (Figure 1).

To start, we installed the MediaWiki framework with the Semantic MediaWiki extension. We then set up a procedure to import all the Gene Wiki pages from Wikipedia and all SNP pages from SNPedia linked to a Gene Wiki gene using a program we created called SyncBot (Figure 1.1a, 1.1b). For the initial import, SyncBot passed each article’s content through the NCBO Annotator Web service to find occurrences of terms from the Human Disease Ontology. The bot then copied the page to the new wiki (Figure. 1.2), and appended the Disease Ontology terms detected in the Annotator to the end of each article (Figure. 1.3), forming semantic links between the gene or SNP and any associated diseases. In addition, the bot appended gene pages with the identifier of each SNP that occurred on the gene. This established the gene-to-disease and gene-SNP-disease links shown in Figure 1.4.

After the initial import, the bot continuously monitors the source wikis for changes to the original articles. When the source content is updated, the bot repeats the import and annotation process with only the changed pages, thus ensuring that the Gene Wiki+ stays up-to-date. (The code for the SyncBot is open source and can be accessed from the Gene Wiki+ software download page [20].)

## Results

Gene Wiki+ seamlessly integrates a multitude of knowledge sources. Both source wikis leverage and filter numerous large public datasets, such as dbSNP, HapMap, OMIM, PDB, PharmGKB and PubMed. Software bots from a distributed developer community continuously update both SNPedia and the Gene Wiki with structured content. SNPedia bots mine the scientific literature to populate new textual wiki content, whereas all textual content on the Gene Wiki is manually entered. All content in both wikis is curated and expanded by a human editor community. All of this information flows into the Gene Wiki+ through a continuous one-way syncing process providing users with up-to-date access to a diverse body of gene and disease related knowledge.

**Figure 2:**
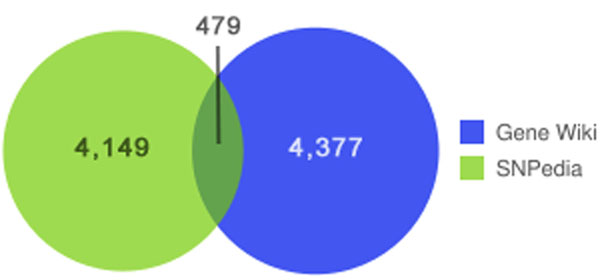
**Overlap of gene-disease associations derived from SNPedia and the Gene Wiki.** 4,149 gene-disease relationships were found using the text of SNPedia articles and 4,377 were found in the text of the Gene Wiki. Only 479 distinct gene-disease pairs were found in both resources.

Overall, the Gene Wiki+ meta-wiki captures 8,047 distinct gene-disease relationships where genes are indicated by NCBI Gene identifiers and diseases are represented with Disease Ontology terms. There are 3,238 distinct genes and 1,060 distinct diseases represented in these gene-disease pairs. SNPedia accounts for 4,149 of the gene-disease pairs via gene-SNP-disease connections, while the Gene Wiki provides 4,377 via direct gene-disease associations. Only 479 (6%) of the gene-disease pairs appear independently in both sources (Figure 2).

The 479 gene-disease pairs in the overlap contained 227 distinct diseases and 376 distinct genes. For example, the gene CYSLTR1 is linked to asthma in the text of the Gene Wiki: “The cysteinyl leukotrienes...] are important mediators of human bronchial asthma” and in the text of the SNPedia article on rs320995 (a SNP found in the CYSLTR1 gene): “subjects without T-allele in SNP rs320995 had 3.1 times higher risk of asthma” [21].

As Figure 2 clearly illustrates, both the Gene Wiki and SNPedia contain substantial amounts of knowledge pertinent to the challenge of finding associations between genes and diseases. The low level of overlap between the gene-disease associations found in these resources indicates the potential value of their combination.

### Gene Wiki+ RDF

One of the key advantages of the Semantic Media Wiki framework is its ability to generate structured exports of the knowledge it contains that adhere to the Resource Description Framework (RDF) standard. This makes it possible to take advantage of the growing collection of tools built on this standard to conduct analysis of the data. Loading the RDF exported from Gene Wiki+ into a triplestore such as Jena, AllegroGraph, or OpenLink Virtuoso makes it possible to execute SPARQL queries, to integrate the data with other RDF resources, and to take advantage of Semantic Web reasoning systems. (SPARQL is the standard query language for RDF.)

As one example, the 479 gene-disease pairs in the overlap between the Gene Wiki and SNPedia can be identified with the following SPARQL query executed in a triplestore.

PREFIX wiki: <http://genewikiplus.org/wiki/Special:URIResolver/>

PREFIX property: <http://genewikiplus.org/wiki/Special:URIResolver/Property-3A> PREFIX rdf: <http://www.w3.org/1999/02/22-rdf-syntax-ns#>

### Inference in Gene Wiki+

Since we imported the Human Disease Ontology class hierarchy into the Gene Wiki+ as MediaWiki categories, the subclass relationships from the ontology are accessible both within the wiki and in the exported RDF. Inside the Gene Wiki+, we can make use of Semantic MediaWiki’s category processing capability to process these hierarchical relationships. For example, we can ask for all genes related to diseases of mental health with the following inline query:

This query produced 237 results - all of which were obtained through inference (i.e. no gene was annotated directly to the term ‘disease of mental health’).

The RDF export process translates MediaWiki categories into simple OWL (Web Ontology Language) classes without logical definitions and sub-category relationships into RDF-Schema subclass relations. As a result, the export contains a mirror of the class hierarchy from the Disease Ontology. This makes it possible to utilize basic reasoning over this hierarchy when processing the exported RDF.

### The Gene Wiki+ as Linked Data

In addition to manipulating the data in Gene Wiki+ with RDF processing tools, the RDF export facilitates integration with other data sources in the Linked Data cloud. All of the articles from the Gene Wiki are linked via owl:sameAs relationships to the equivalent entities in DBPedia [22]. As DBpedia forms one of the primary hubs of the Linked Data cloud [23], this connection ties the Gene Wiki+ meta wiki directly into the rest of the Semantic Web. Aside from DBpedia integration, all of the categories brought in from the Disease Ontology are marked with OWL:equivalentClass relationships to the corresponding classes in the Disease Ontology as indicated by their OBO URIs. For example, the Gene Wiki+ category for Vulvar keratoacanthoma-like carcinoma is marked as equivalent to the entity in the Disease Ontology identified by the URI http://purl.obolibrary.org/obo/DOID_7408. By supplying stable URIs, facilitating data access as RDF, and maintaining clear relationships to other Semantic Web resources, the Gene Wiki+ meta-wiki is ready for use in RDF-based applications that facilitate interaction with data drawn dynamically from multiple sources.

### Enhancements to the user experience

**Figure 3:**
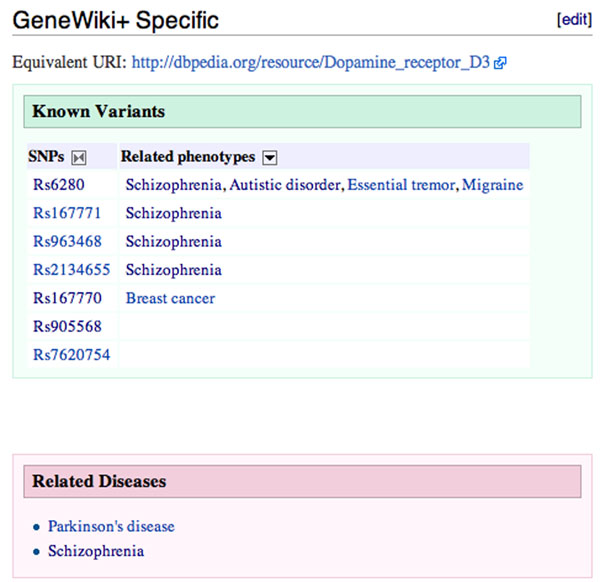
**Enhancements to the article on Dopamine receptor D3**. The new ‘Gene Wiki+ Specific’ section, shown on all gene articles in GeneWiki+, includes: the link to the equivalent article in DBpedia, the table of known variants for the gene with related phenotypes, and the related diseases identified in the article text. Note the differences between the disease associations found in the related SNPs and the associations found in the text for this article.

While the aggregation of data from multiple sources in a queryable, structured form is useful for computational scientists, few ‘end-user’ biologists can be expected to enter SPARQL queries or even queries in the Semantic Media Wiki syntax. For the majority of users, the value of a meta-wiki such as this is in the direct improvements to the individual articles that they will discover while browsing. Hence we made three specific additions to the visible areas of the meta-wiki articles. First, we added a ‘known variants’ table to all of the gene articles. This table presents SNPs related to the gene described in the article and phenotypes related to those SNPs drawn from the data gathered from SNPedia. Next, we added a table displaying diseases found in the text of the Gene article. Figure 3 shows these enhancements to the article on Dopamine receptor D3.

**Figure 4:**
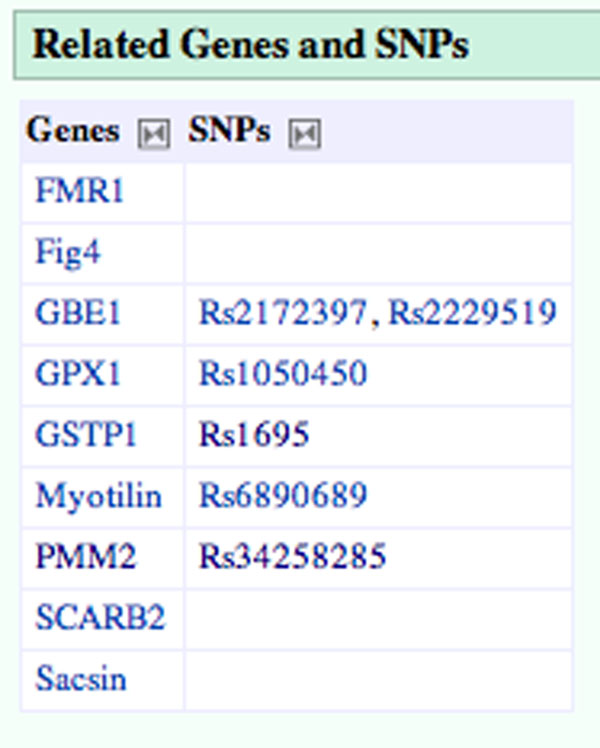
**Enhancements to the article on peripheral neuropathy**. This figure provides an example of the ‘related genes and SNPs’ boxes added to the disease articles from data collected from both SNPedia and the Gene Wiki. It shows some of the genes and SNPs linked to peripheral neuropathy. Where no SNPs are indicated, the gene is related directly to the disease in the gene’s article text.

In addition to the enhancements to the gene articles, we added a ‘related genes and SNPs’ table to the disease articles. (The disease articles were brought in from Wikipedia as part of the Gene Wiki import. Where no disease article existed in Wikipedia, we created a stub from the Disease Ontology definition.) This table presents genes and SNPs that are linked to the disease either in the text of a Gene Wiki article or through genetic associations found in SNPedia. Figure 4 shows how the article on peripheral neuropathy has been expanded with a section detailing related genes as well as related SNPs on these genes.

## Discussion

The low amount of overlap between the gene-disease relationships found in the Gene Wiki and the gene-SNP-disease relationships from SNPedia was surprising. Based on a review of the meta-wiki assembly process and manual inspections of the data, we could not determine any particular systematic driver of the observed differences. It appears that the two data sources simply contain different data. As one example, the text of the Gene Wiki article on the Dopamine receptor D3 gene contains a reference to its relationship to Parkinson’s disease, backed by [24], that is not captured on any of its seven related SNPs. At the same time, the related SNP Rs167770 indicates a relationship to breast cancer, supported by [25], that is not represented in the text of the Gene Wiki article (Figure 3). While this is just an anecdote, it is indicative of the underlying trend in the data as presented in Figure 2.

There are many reasons why a particular gene might be associated with a disease in a Gene Wiki article that do not implicate a particular SNP. For example, genes may be involved in pathways known to be important to disease pathogenesis or to the body’s immune response while there may not be any known SNPs associating that gene with that disease. On the other hand, if a SNP linked to a particular gene is related to a disease, it seems likely that the gene-disease connection should be made in the text of the article about the gene. In the latter case, the gene-SNP-disease associations provide excellent starting points for potential expansions of Gene Wiki articles.

It is important to note that there is no well-accepted standard reference database for structuring and curating gene-disease associations. While several databases provide information relating genes to diseases e.g. PharmGKB, Online Mendelian Inheritance in Man (OMIM), and UniProt (see ‘involvement in disease’ attribute), the information in each one is different, each uses their own vocabulary and none has emerged as a unifying standard. In this work we seek not to replace such curated resources, but to provide a crowd-sourced complement to them.

One of the weaknesses of the approach used to build this meta-wiki is that it represents a one-way sync. If editors make changes to the articles in the meta-wiki, there is currently no automated mechanism for migrating those changes back to the articles in the original wikis. While one option is to let these meta-wikis evolve independently of their parents, a better approach might be to establish mechanisms through which edits made to a meta-wiki article could flow back into the articles used to create them. Such a mechanism would effectively extend the reach of the source wikis - both in terms of exposing their contents and of acquiring more editors. There are tools emerging that will make this possible. For example, the Distributed Semantic Media Wiki system is an extension that enables the creation of a network of Semantic Media Wiki servers that share common semantic wiki pages [26]. With such a system in place, we might imagine that meta-wikis like the one discussed here could serve not only as new integrated resources for consuming information but also new points for users to contribute information back to the community collection.

## Conclusions

We have demonstrated how a high-level linking of genes and diseases can be accomplished through the meta-wiki approach, but we have not touched on the deeper, more difficult question of how these genes are linked to these diseases. To address this complex challenge, the work of thousands of specialists needs to be assembled into integrated wholes that can be understood and used to drive action. The topic-focused wikis emerging in different areas of biology represent one step of this process of collaborative knowledge synthesis. Looking forward, meta-wikis such as the one presented here offer the potential to go one step further - to help unearth and present the latent relationships that exist between different concepts and different communities.

## Competing interests

The authors declare they have no competing interests.

## Authors' contributions

BMG, SL, and AIS had the initial idea. ELC and SL produced the code for the meta-wiki mashup server. BMG conducted the data analysis and drafted a preliminary version of the manuscript. All authors read, contributed to, and approved the final manscript.
